# Emergency orchidectomy: a rare complication of diverticular colovesical fistula

**DOI:** 10.1093/jscr/rjag164

**Published:** 2026-03-17

**Authors:** Mashaal Hamayun, Mina Sarofim, Andrew Gilmore

**Affiliations:** Department of Colorectal Surgery, Liverpool Hospital, Liverpool, NSW 2170, Australia; Innovation, Surgical Teaching and Research Unit, Liverpool Hospital, Liverpool, NSW 2170, Australia; Faculty of Medicine and Health, University of Sydney, Sydney, NSW 2006, Australia; Department of Colorectal Surgery, Liverpool Hospital, Liverpool, NSW 2170, Australia; Innovation, Surgical Teaching and Research Unit, Liverpool Hospital, Liverpool, NSW 2170, Australia; Faculty of Medicine and Health, University of Sydney, Sydney, NSW 2006, Australia; Department of Colorectal Surgery, Liverpool Hospital, Liverpool, NSW 2170, Australia; School of Medicine, Western Sydney University, Penrith, NSW 2751, Australia; Faculty of Medicine and Health Sciences, Macquarie University Hospital, Macquarie Park, NSW 2109, Australia

**Keywords:** colovesical fistula, diverticulitis, epididymo-orchitis, pneumaturia, surgical resection

## Abstract

Acute epididymo-orchitis (AEO) is an uncommon complication of colovesical fistula (CVF). We present the first documented case of a patient requiring emergency orchidectomy for recurrent AEO, complicated by intratesticular necrosis and a scrotal wall abscess, secondary to an undiagnosed diverticular CVF. Definitive management involved a laparoscopic high anterior resection, employing trans anal natural orifice specimen extraction, to cure the fistula. This case underscores the importance of maintaining a high index of suspicion for rare anatomical abnormalities such as CVF in patients presenting with recurrent bacterial AEO, across specialties for both the urologist and colorectal surgeon. Early and thorough investigation is crucial to avoid the severe complications of untreated diverticular disease—particularly in individuals with significant intra-abdominal adiposity, where classic signs of peritoneal inflammation may be absent. Delayed diagnosis can lead to serious outcomes, including testicular loss and life-threatening sepsis.

## Introduction

Acute epididymo-orchitis (AEO) is a rare consequence of colovesical fistula (CVF). We present the first case of a patient requiring an emergency orchidectomy, for recurrent epididymo-orchitis complicated by intratesticular necrosis and scrotal wall abscess, secondary to an undiagnosed diverticular CVF. The patient subsequently required a laparoscopic high anterior resection to cure the diverticular fistula.

## Case report

A 40-year-old male presented to the emergency department of a tertiary hospital with a third episode of left AEO over a 12-month period. In addition to testicular pain, his symptoms included new pneumaturia, faeculuria, and haematuria. On examination, he was febrile with a swollen, indurated, and tender left testis. His past medical history included type 2 diabetes mellitus, morbid obesity (body mass index [BMI] 51.9 kg/m^2^), and 10 pack-year smoking; there were no previous surgical or endoscopic procedures.

**Figure 1:**
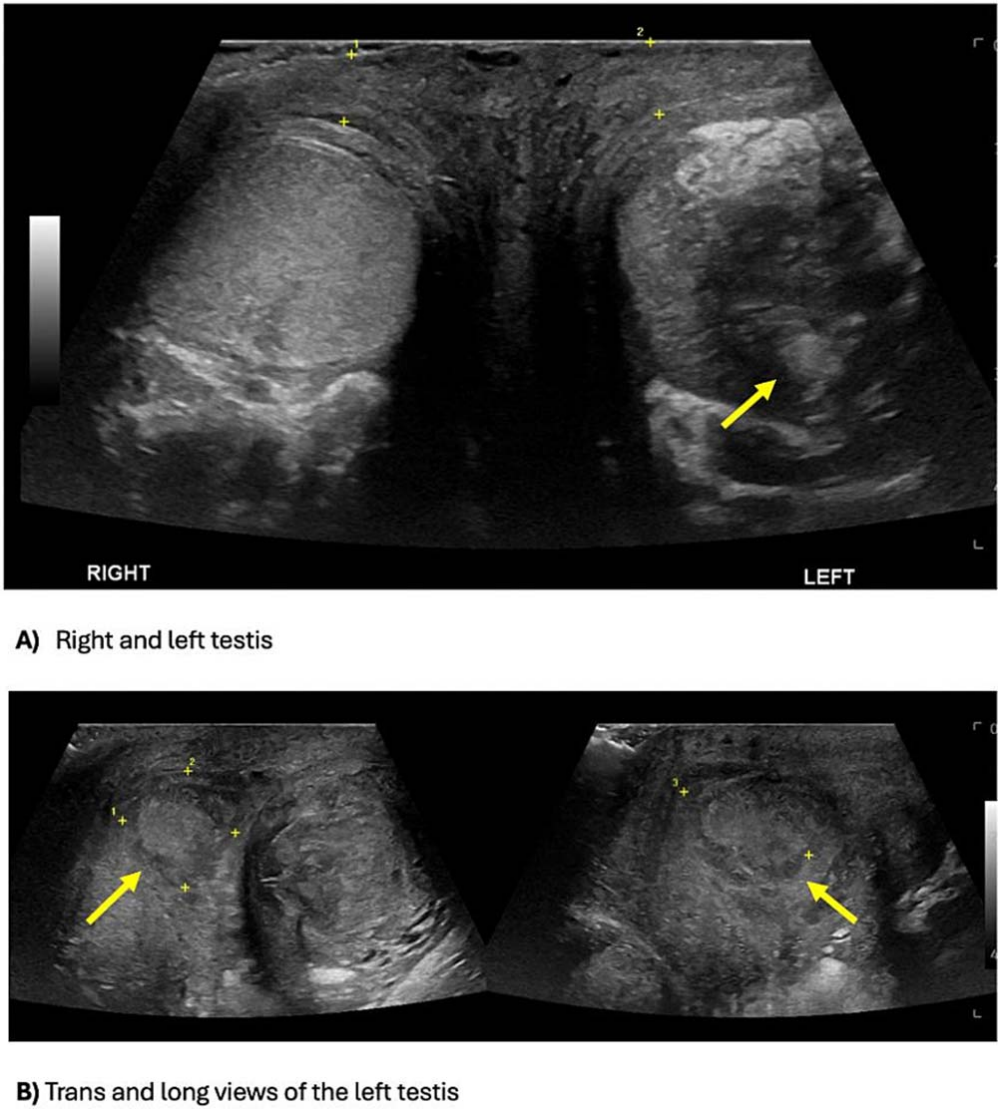
Scrotal ultrasound demonstrating an ill-defined and heterogeneous left testis with a central hypoechoic part of decreased vascularity suggestive of an abscess, versus the homogenous and normal vascularity right testis (A). The testicular abscess extends medially through the tunica albuginea, causing a scrotal wall abscess (B).

**Figure 2:**
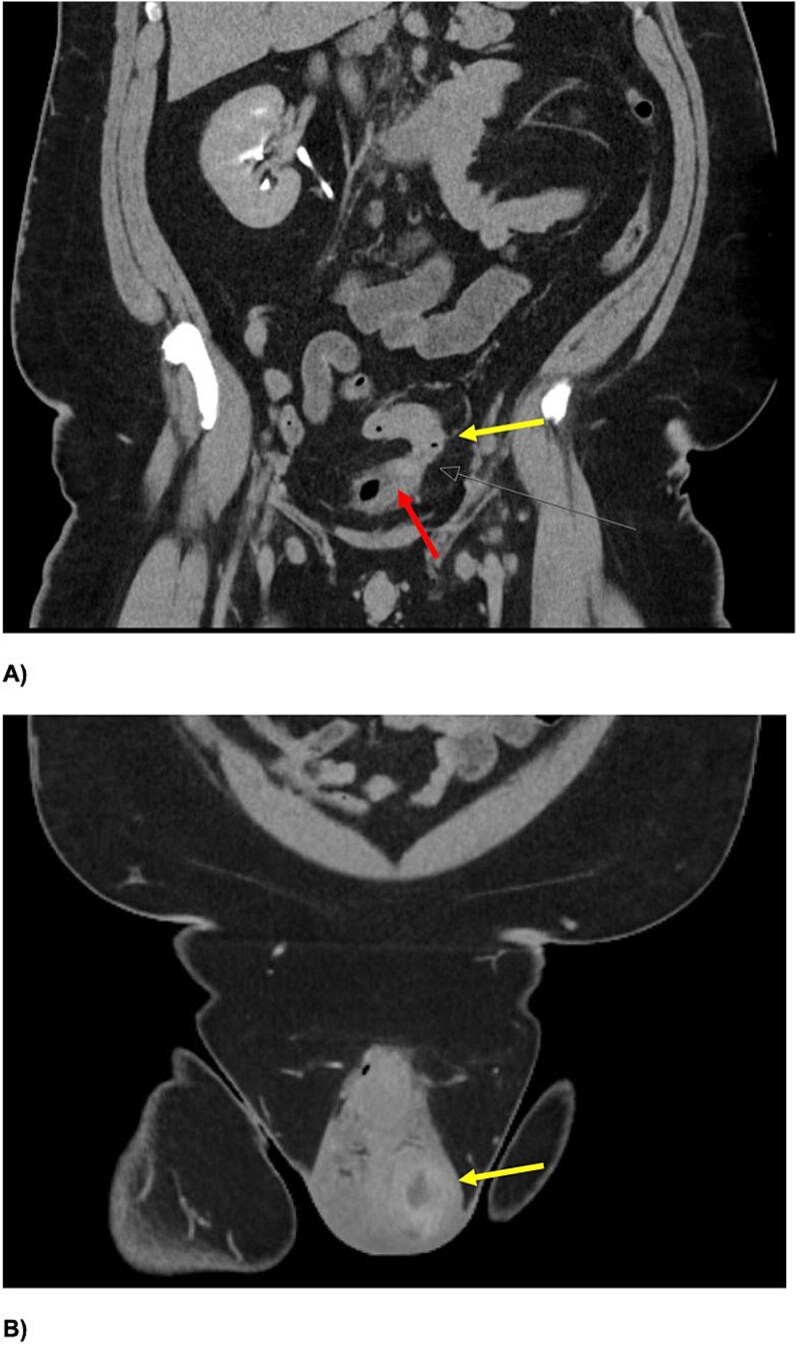
Coronal slices of CT IVP (A) demonstrating a colovesical fistula between the inferior wall of the proximal sigmoid colon (yellow arrow (A)) and superior aspect of the urinary bladder (red arrow). There is intravesical gas with eccentric irregular anterior bladder wall thickening, and mild perivesical fat stranding. An enlarged left testis, with central hypodensity, and left testicular-scrotal abscess is also demonstrated (B).

Inflammatory markers were mildly elevated—white cell count 12 × 10^9^/l, C-reactive protein 30 mg/l. Scrotal ultrasound demonstrated a left intratesticular abscess, ruptured tunica albuginea, and suspected ischaemia (Fig. 1). Ordered due to recurrent AEO, a computed tomographic (CT) intravenous pyelogram revealed a fistula between the sigmoid colon and dome of the bladder, secondary to diverticular disease (Fig. 2). Urine cultures from his last admission for epididymo-orchitis grew multi-resistant *Escheria coli* and *Enterococcus faecium*.

**Figure 3:**
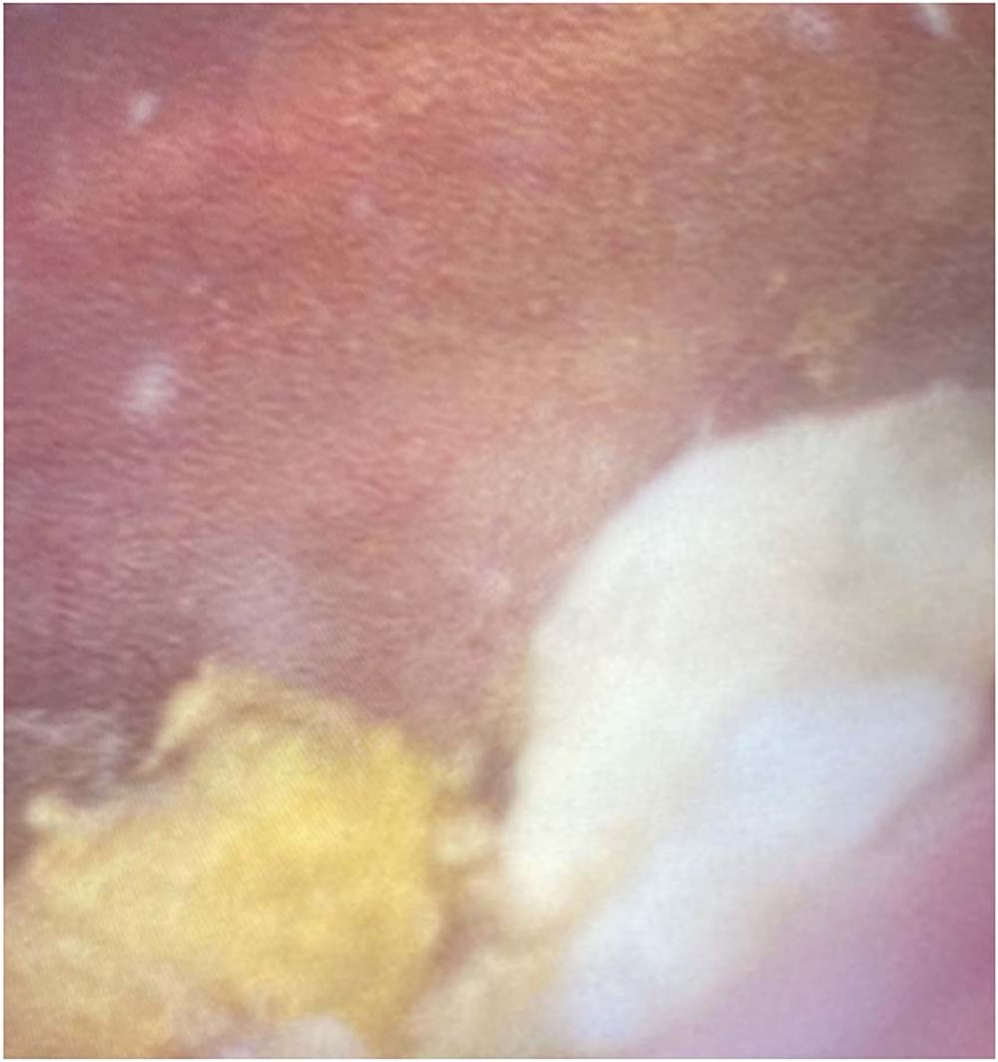
Cystoscopy image demonstrating significant faeculant debris within the bladder mucosa.

The patient was managed surgically with scrotal exploration and left orchidectomy. Intra-operatively the superior pole of the testis was necrotic, with pus within the ruptured tunica albuginea. Cystoscopy demonstrated faeculant debris within the bladder (Fig. 3). Post-operatively he recovered well and was discharged on day two with a prolonged course of intravenous antibiotic therapy in the community.

Four months later, after 11 kg of weight loss (BMI 47.9 kg/m^2)^), he underwent elective colonoscopy and laparoscopic anterior resection, employing trans anal natural orifice specimen extraction (NOSE). He had an uncomplicated recovery and discharge on Day 5. Histopathology confirmed diverticular disease with abscess.

## Discussion

Acute bacterial epididymo-orchitis is an occasional urological presentation in men, defined as inflammation of the epididymis and testis [1]. Often occurring unilaterally, hallmark symptoms are testicular pain and swelling [1]. Aetiology is most commonly urinary tract-associated Gram-negative organisms in older men with bladder out flow obstruction, due to benign prostatic hyperplasia. Other causes include sexually transmitted organisms such as *Chlamydia trachomatic* or *Neisseria gonorrhoea*, usually in men aged under 35 [2]. Timely and effective antibiotic management is important due to potential long-term consequence such as infertility, abscess, infarction, and strictures [2].

Patients with urinary tract associated AEO are recommended investigations for structural abnormalities or genito-urinary obstruction [3, 4]. In the present case, such investigation revealed AEO secondary to an undiagnosed CVF, an extremely rare phenomenon. Only one prior case report, to our knowledge, describes recurrent AEO as the presenting complaint for complicated diverticular disease [5]. This is also the first case where a patient has required emergency orchidectomy for severe CVF, reflecting the serious consequences that can arise from a delayed diagnosis. The finding was somewhat unexpected as the patient had no clinical history suggestive of diverticular disease. Clinicians should consider however that extreme intrabdominal adiposity, as our patient had, can prevent symptomatic inflammation of the parietal peritoneum (in a manner analogous to asymptomatic abscess from retrocaecal appendicitis [6]). Cross-speciality awareness is thus also crucial for early recognition and collaboration between the urologist and colorectal surgeon.

CVF is an abnormal connection between the urinary bladder and colon [7]. It is most commonly a complication of diverticular disease, where increased intraluminal pressure with abnormal peristalsis results in diverticular perforation. The consequent abscess or phlegmon will then erode into the bladder creating a fistulous tract [3]. Other causes of CVF include malignancy, inflammatory bowel disease, iatrogenic injury, radiation, and tuberculosis [4, 7]. The absence of a uterus in males—which divides the colon from the bladder—means there is a greater preponderance of CVF in men, with an average age of 55–75 years at presentation [7].

Clinically CVF is pathognomonically characterized by pneumaturia (70%–90%) and faecaluria (50–70%) [4]. Recurrent urinary tract infections should also raise suspicion for a CVF. While there is no gold standard diagnostic algorithm, investigations are useful for anatomical delineation/visualization, identifying strictures and determining underlying aetiology, particularly in cases of malignancy, which in turn guides operative management [4, 8]. Computed tomography will often reveal air or oral contrast in the bladder, stranding of the colon/bladder, strictures, or malignancy [4]. Cystoscopy findings are usually non-specific—erythema, oedema, congestion—with failure to detect CVF in over 50% of cases [4]. Detection rates of CVF in colonoscopy are also sorely variable, ranging from 0% [8, 9] to 9% [10] to as high as 55% [11]; however, as stated above, it remains helpful in characterizing intestinal pathology, and excluding suspected malignancy [4].

The high risks of complications such as progression in cases of malignant fistulas, and urosepsis, means operative management is recommended for CVF [9]. Non-operative management is thus reserved for the surgically unfit and includes antibiotics, catheter drainage, and rarely insertion of a covered colonic stent [4, 9]. Definitive surgical resection of the fistula tract and involved colon and bladder may be performed laparoscopically; this has the advantages of shorter length of stay, less opioid usage, and similar complication rates to open procedures [3]. It may be achieved as a single or two-stage operation, with or without the use of a stoma, followed by reversal, which is surgeon dependent. In our case, a trans anal NOSE approach was selected to reduce the morbidity associated with traditional abdominal wall specimen extraction incisions, which can be complicated by pain, surgical site infections, and long-term incisional hernias [12]. This innovative approach is associated with excellent perioperative outcomes, thus highlighting its feasibility as an adjunct in operative management of diverticular disease [12].

## Conclusion

This case represents the first reported instance of emergency orchidectomy for recurrent epididymo-orchitis secondary to an undiagnosed diverticular CVF. It highlights the critical need to investigate recurrent bacterial AEO for underlying anatomical abnormalities, even in the absence of prior suggestive history. This is important in obese patients, where significant intra-abdominal adiposity may mask classical symptoms. Early recognition, timely surgical intervention, and collaboration between surgical specialties are thus essential to prevent serious complications of such as testicular loss and sepsis and achieve definitive management of complicated diverticular disease.

## References

[1] Street EJ, Wilson JD. Acute epididymo-orchitis. Medicine 2014;42:338–40. 10.1016/j.mpmed.2014.03.012

[2] Norton SM, Saies A, Browne E et al. Outcome of acute epididymo-orchitis: risk factors for testicular loss. World J Urol 2023;41:2421–8. 10.1007/s00345-023-04500-1 37452204 PMC10465682

[3] Keller-Biehl L, Yu KR, Smith-Harrison L et al. Colovesical fistula: a 28 year experience at a major United States department of veterans affairs medical center. Surg Pract Sci 2022;11:100100. 10.1016/j.sipas.2022.100100 39845169 PMC11749922

[4] Golabek T, Szymanska A, Szopinski T et al. Enterovesical fistulae: aetiology, imaging, and management. Gastroenterol Res Pract 2013;2013:1–8. 10.1155/2013/617967 PMC3857900 24348538

[5] Tay JY, Hayes I, Fisher T et al. Complicated diverticular disease presenting as recurrent epididymo-orchitis: a case report. ANZ J Surg 2021;91:E703–5. 10.1111/ans.16751 33734549

[6] Fanning DM, Barry M, O’Brien GC et al. Perforated retrocaecal appendix presenting as right lumbar abscess. Ir J Med Sci 2007;176:125–8. 10.1007/s11845-007-0040-z 17516131

[7] Waack A, Ranabothu M, Patel NJ. Epididymo-orchitis secondary to colovesical fistula. Urol Case Rep 2022;45:102281. 10.1016/j.eucr.2022.102281 36438455 PMC9685279

[8] Najjar SF, Jamal MK, Savas JF et al. The spectrum of colovesical fistula and diagnostic paradigm. Am J Surg 2004;188:617–21. 10.1016/j.amjsurg.2004.08.016 15546583

[9] Garcea G, Majid I, Sutton CD et al. Diagnosis and management of colovesical fistulae; six-year experience of 90 consecutive cases. Color Dis 2006;8:347–52. 10.1111/j.1463-1318.2005.00928.x 16630242

[10] Melchior S, Cudovic D, Jones J et al. Diagnosis and surgical management of colovesical fistulas due to sigmoid diverticulitis. J Urol 2009;182:978–82. 10.1016/j.juro.2009.05.022 19616793

[11] Kavanagh D, Neary P, Dodd JD et al. Diagnosis and treatment of enterovesical fistulae. Color Dis 2005;7:286–91. 10.1111/j.1463-1318.2005.00786.x 15859969

[12] Sarofim M, Mui J, Cartmill J et al. Natural orifice specimen extraction for diverticular disease: technique, outcomes and role of inflammatory markers. Surg Endosc 2025;39:4245–52. 10.1007/s00464-025-11803-4 40394329 PMC12222262

